# Association Between Plasma Amyloid-β and Neuropsychological Performance in Patients With Cognitive Decline

**DOI:** 10.3389/fnagi.2021.736937

**Published:** 2021-10-25

**Authors:** Gyihyaon Yun, Hye Jin Kim, Hyug-Gi Kim, Kyung Mi Lee, Il Ki Hong, Sang Hoon Kim, Hak Young Rhee, Geon-Ho Jahng, Sung Sang Yoon, Key-Chung Park, Kyo Seon Hwang, Jin San Lee

**Affiliations:** ^1^Department of Neurology, Kyung Hee University Hospital, Kyung Hee University College of Medicine, Seoul, South Korea; ^2^Department of Clinical Pharmacology and Therapeutics, Kyung Hee University College of Medicine, Seoul, South Korea; ^3^Department of Radiology, Kyung Hee University Hospital, Kyung Hee University College of Medicine, Seoul, South Korea; ^4^Department of Nuclear Medicine, Kyung Hee University Hospital, Kyung Hee University College of Medicine, Seoul, South Korea; ^5^Department of Otorhinolaryngology, Head and Neck Surgery, Kyung Hee University Hospital, Kyung Hee University College of Medicine, Seoul, South Korea; ^6^Department of Neurology, Kyung Hee University Hospital at Gangdong, Kyung Hee University College of Medicine, Seoul, South Korea; ^7^Department of Radiology, Kyung Hee University Hospital at Gangdong, Kyung Hee University College of Medicine, Seoul, South Korea

**Keywords:** Alzheimer’s disease, amyloid-β1-40, amyloid-β1-42, memory, blood-based biomarker, cognitive decline, neuropsychological performance

## Abstract

**Objective:** To investigate the association between plasma amyloid-β (Aβ) levels and neuropsychological performance in patients with cognitive decline using a highly sensitive nano-biosensing platform.

**Methods:** We prospectively recruited 44 patients with cognitive decline who underwent plasma Aβ analysis, amyloid positron emission tomography (PET) scanning, and detailed neuropsychological tests. Patients were classified into a normal control (NC, *n* = 25) or Alzheimer’s disease (AD, *n* = 19) group based on amyloid PET positivity. Multiple linear regression was performed to determine whether plasma Aβ (Aβ_40_, Aβ_42_, and Aβ_42/40_) levels were associated with neuropsychological test results.

**Results:** The plasma levels of Aβ_42/40_ were significantly different between the NC and AD groups and were the best predictor of amyloid PET positivity by receiver operating characteristic curve analysis [area under the curve of 0.952 (95% confidence interval, 0.892–1.000)]. Although there were significant differences in the neuropsychological performance of cognitive domains (language, visuospatial, verbal/visual memory, and frontal/executive functions) between the NC and AD groups, higher levels of plasma Aβ_42/40_ were negatively correlated only with verbal and visual memory performance.

**Conclusion:** Our results demonstrated that plasma Aβ analysis using a nano-biosensing platform could be a useful tool for diagnosing AD and assessing memory performance in patients with cognitive decline.

## Introduction

Alzheimer’s disease (AD), a neurodegenerative disease, is the most common cause of cognitive impairment and dementia (Jack et al., 2018). The pathophysiology of AD is characterized by extracellular amyloid-β (Aβ) plaques and intracellular tau deposition in the brain (Pereira et al., 2021). Growing evidence suggests that Aβ deposition in the brain initiates AD by inducing a chain of events involving the accumulation of toxic tau, which consequently leads to downstream neurodegeneration (Jack et al., 2013, 2014; Pereira et al., 2021). Pathophysiological changes in AD progress over many years without clinical symptoms or dementia; therefore, early detection of Aβ pathology has clinical significance for the deterioration of cognitive function and the occurrence of dementia.

The detection of Aβ pathology using cerebrospinal fluid (CSF) analysis and *in vivo* amyloid positron emission tomography (PET) scans has shown a high correlation with postmortem findings (Palmqvist et al., 2015; Hansson et al., 2018); however, their use in clinical settings is limited. Considering the advantages in terms of cost, reduced invasiveness, and repeated measurement ability, there is a need for the development of blood-based AD biomarkers. However, since Aβ is present at extremely low levels in the blood (on the scale of pg/mL) (Risacher et al., 2019), a more accurate measurement method with higher sensitivity and selectivity is required for the development of more precise blood-based AD biomarkers. Aβ is produced by proteolytic cleavage of amyloid precursor proteins into a monomeric form by β- and γ-secretases, which are transformed into oligomeric and fibrillar forms, and finally into amyloid plaques (O’Brien and Wong, 2011). With advances in measurement technology, it has become possible to measure various forms of Aβ; however, published results thus far appear to be fairly conflicting, and it has been reported that several factors influence the measurement of Aβ in the blood (Wang et al., 2020).

We previously developed a sensing platform capable of analyzing biomolecules at levels in the range of fg/mL with high accuracy (Kim et al., 2019). Our biosensing platform is manufactured via micro/nano-fabrication technology and detects changes in electrical signals, particularly impedance changes, to quantify fg/mL of biomolecules. Additionally, we quantified the level of plasma Aβ, including the monomeric form, more accurately through the filtration effect induced by the dielectrophoresis (DEP) force. Using this method, we overcame the limitations of Aβ monomer quantification in plasma by mitigating the non-specific binding of matrix factors, and eventually more accurately detected Aβ levels in the blood.

Previous studies have revealed that the oligomeric form of Aβ has neuronal toxicity associated with AD pathology as well as synaptic dysfunction (Lacor et al., 2007; Shankar et al., 2008; Viola and Klein, 2015). In addition, elevated levels of Aβ oligomers in the CSF or blood are negatively correlated with neuropsychological performance in patients with AD (Santos et al., 2012; Wang-Dietrich et al., 2013; Meng et al., 2019). To the best of our knowledge, there have been no studies evaluating the association between the level of plasma Aβ and neuropsychological performance, including monomeric Aβ. Thus, the present study aimed to investigate the association between the level of plasma Aβ (Aβ_40_, Aβ_42_, and Aβ_42/40_) and neuropsychological performance in patients with cognitive decline, classified according to amyloid PET positivity using a highly sensitive nano-biosensing platform. Considering that neuropsychological performance in all cognitive domains, including attention, language, visuospatial, memory, and frontal/executive functions, are impaired in patients with AD (Scheltens et al., 2021), we hypothesized that the level of plasma Aβ would be negatively correlated with neuropsychological performance of all cognitive domains in patients with cognitive decline.

## Materials and Methods

### Reagents and Materials

All reagents and materials are listed in Supplementary Material.

### Study Participants

We prospectively recruited 44 individuals who underwent plasma Aβ analysis, high-resolution 3.0-T brain magnetic resonance imaging (MRI), ^18^F-florbetaben PET, and detailed neuropsychological testing (Ahn et al., 2010). The participants comprised 20 patients with subjective cognitive decline (SCD), 7 with amnestic mild cognitive impairment (aMCI), and 17 with probable AD dementia, all of whom had been clinically diagnosed at the Memory Disorders Clinic of Kyung Hee University Hospital (Seoul, Korea) between December 2018 and September 2019. Patients with SCD had subjective memory decline but no objective cognitive dysfunction in any cognitive domain in the detailed neuropsychological tests (Jessen et al., 2014). Patients with aMCI were diagnosed according to the Petersen criteria (Petersen, 2004) with modifications that have been described elsewhere (Seo et al., 2009). Patients with probable AD dementia met the criteria proposed by the National Institute of Neurological and Communicative Disorders and Stroke and the AD and Related Disorders Association (McKhann et al., 1984). Brain MRI confirmed the absence of structural lesions, including cerebral hemorrhage or infarction, hippocampal sclerosis, brain tumors, traumatic encephalomalacia, and vascular malformation. The exclusion criteria included a history of psychological disease, stroke, brain surgery, seizure, head trauma, severe cerebral white matter hyperintensities defined by the modified Fazekas scale (Seo et al., 2009), or current systemic medical diseases that could affect cognition.

Laboratory tests were conducted for all participants to eliminate any other causes of cognitive impairment. These tests included complete blood counts, vitamin B_12_ levels, folate levels, metabolite profiles, thyroid function tests, and syphilis serology. Genomic DNA was extracted from whole blood samples for apolipoprotein E (*APOE*) genotyping according to the manufacturer’s instructions (QIAGEN GmbH, Hilden, Germany).

### Standard Protocol Approval, Registration, and Patient Consent

Written informed consent was obtained from all participants (surrogates or legally authorized representatives if the patient did not have the cognitive ability to provide consent to study participation) prior to inclusion in the study. The study was approved by the Institutional Review Board (IRB) of Kyung Hee University Hospital (IRB No. 2018-01-023).

### Plasma Acquisition and Sampling Protocol

To demonstrate an association between the level of plasma Aβ and neuropsychological performance, we collected approximately 1 mL of blood from each participant in an anticoagulant-treated heparin vacutainer tube (sodium heparin vacutainer, cat. no. 367874; BD Biosciences, Franklin Lakes, NJ). Human plasma was then separated from the blood using an ultra-centrifuge (3,000 rpm at 4°C for 15 min) and transferred into a polypropylene (PP) tube. Finally, the plasma was mixed with 1 × protease inhibitor (Version 09, complete mini-ethylenediaminetetraacetic acid-free) in 10 mM phosphate-buffered saline (PBS), and then approximately 1 mL was aliquoted into fresh 1.5-mL PP tubes. The aliquoted samples were stored frozen (−80°C) and diluted 1/100 in 1 mM PBS buffer immediately prior to plasma analysis.

### Fabrication and Modification of the Sensing Platform

The sensing platform was fabricated via a microelectromechanical system (MEMS) (Kim et al., 2016). First, to fabricate the biosensing platform, layers of silicon dioxide (SiO_2_) and 300/1500 Å tantalum/platinum (Ta/Pt) were deposited on a 4-inch silicon wafer. Then, the Ta/Pt layers were patterned via photolithography and reactive ion etching (RIE). The single sensing platform consisted of six pairs of interdigitated microelectrodes (IMEs) designed with 30 pairs of electrode fingers with 5-μm spacing and 300-μm length.

Meanwhile, two types of antibodies, 11A50-B10 and 12F4, were immobilized on the surface sensor to analyze Aβ_40_ and Aβ_42_, respectively. The surface of the sensor was cleaned and chemically activated by sequential treatment with piranha cleaning solution (H_2_SO_4_:H_2_O_2_ = 4:1), 1% 3-(ethoxydimethylsilyl) propylamine (APMES), and *N*-(3 -dimethyl aminopropyl)-*N*-ethylcarbodiimide hydrochloride /*N*-hydroxysuccinimide (EDC/NHs) cross-linking solution. Then, 10 μL of antibody solution was dropped on the surface of the sensor to immobilize the antibody.

### Immunoassay and Signal Analysis

To quantify Aβ, a polydimethylsiloxane (PDMS) microfluidic channel fabricated through the MEMS process was attached to the sensor surface, and 10 μL of Aβ solution diluted into 1 mM PBS and standard human plasma was injected into the channel using a syringe (Kim et al., 2016). Then, using a DG40662 series waveform generator (Rigol Technologies Inc., Beaverton, OR), an alternating current (AC) voltage with an intensity of 0.5 mV and frequency of 60 MHz was applied to encourage a reaction between the antibody and Aβ. This reaction was carried out for 20 min at 25°C, and unbound Aβs were washed with 1 mM PBS after the reaction. The reaction was quantified by comparing the electrical signals (ΔZ) measured before and after the Aβ reaction (ΔZ_before_ and ΔZ_after_, respectively) as follows:


$$\text{|}\Delta \text{Z| }\left(\%\right)=|\frac{{|\text{Z}}_{\text{after|}}-|{\text{Z}}_{\text{before|}}}{|{\text{Z}}_{\text{before|}}}|\times 100$$

Similar to the procedure for quantification of Aβ in PBS and standard plasma, Aβ was quantified in clinical samples to diagnose AD. A single clinical sample was quantified using three sensing platforms (i.e., 18 single IME sensors), and the average of 18 IME signals was used as the final output signal due to the Aβ response in human plasma.

### Neuropsychological Testing and Clinical Assessments

All participants underwent detailed neuropsychological tests using the standardized Seoul Neuropsychological Screening Battery (Ahn et al., 2010; Kang et al., 2019). The battery contains tests for attention (Digit Span Forward and Backward), language [Korean version of the Boston Naming Test (K-BNT)], visuospatial function [Rey-Osterrieth Complex Figure Test (RCFT); copying], verbal and visual memory [Seoul Verbal Learning Test (SVLT) and RCFT; immediate and 20-min delayed recall, and recognition], and frontal/executive function [phonemic and semantic Controlled Oral Word Association Test (COWAT) and the Stroop Test; word and color reading]. Age- and education-specific norms for each test based on 447 cognitively normal individuals were used for comparison. Z-scores lower than −1.0 standard deviation (SD) of the age- and education-adjusted norms were considered abnormal. We also used the Mini-Mental State Examination.

### Brain Magnetic Resonance Imaging

Structural three-dimensional (3D) T1-weighted images (T1WIs) were acquired using a 3.0-T MRI system (Achieva TX, Philips Medical Systems, Best, The Netherlands) equipped with a 32-channel sensitivity-encoding head coil and multi-transmit technology. The T1WI sequence used was a magnetization-prepared rapid-acquisition gradient-echo sequence with the following parameters: relaxation time = 9.0 ms, echo time = 5.0 ms, flip angle = 8°, field-of-view = 256 × 256 mm^2^, and voxel size = 1 × 1 × 1 mm^3^.

### ^18^F-florbetaben Positron Emission Tomography/CT Acquisition and Analysis

3D static PET images were acquired 90 min after intravenous injection of 296 MBq of 18F-florbetaben using a Gemini TF 16 PET/CT scanner (Philips Healthcare, Cleveland, OH). CT images were acquired from the vertex to the skull base (50 mAs; 120 kVp; slice 2 mm), and 10-mm PET images were immediately acquired over the same region. We used the ordered-subset expectation maximization algorithm (iteration = 3 and subset = 33) to reconstruct 3D PET images in a 128 × 128 × 20 matrix with a voxel size of 2 × 2 × 2 mm^3^.

All amyloid PET images were visually assessed and dichotomized as amyloid-positive or amyloid-negative after being reviewed by nuclear medicine physicians who were blinded to the participants’ information. Amyloid PET findings were considered positive when the brain amyloid-plaque load (BAPL) score (Barthel et al., 2011) was 2−3, based on visual assessment. Regarding regional cortical tracer uptake (RCTU), image evaluators used the RCTU scoring system (RCTU 1, no tracer uptake; RCTU 2, moderate tracer uptake; and RCTU 3, pronounced tracer uptake) in the lateral temporal cortex, frontal cortex, posterior cingulate cortex/precuneus, and parietal cortex. In terms of RCTU and BAPL correspondence, an RCTU score of 1 in each brain region was considered to be identical to a BAPL score of 1. An RCTU score of 2 in at least one brain region and no score of 3 was considered equivalent to a BAPL score of 2. An RCTU score of 3 in any of the four brain regions was considered a BAPL score of 3. Amyloid-negative status was given to participants with a BAPL score of 1, while amyloid-positive status was given to those with BAPL scores of 2−3.

We also conducted a quantitative amyloid PET analysis using the cortical standardized uptake value ratio (SUVR) in all PET scans. Based on a previous study (Bullich et al., 2017), an SUVR cut-off of ≥ 1.43 was selected as the amyloid positivity criterion. In this study, our visual assessment strongly corresponded with the binarized global ^18^F-florbetaben PET binding evaluations, as the comparison of the two methods resulted in a high accuracy of 100% [25 participants were amyloid-negative (20 patients with SCD and 5 with aMCI), and 19 were amyloid-positive (2 patients with aMCI and 17 with AD dementia)].

### Pattern Analysis of Aβ Accumulation in the Brain

Pattern analysis was performed with MRI and amyloid PET images to visually estimate the relative levels of plasma Aβ_40_ and Aβ_42_ in the brain. First, cortical and subcortical brain regions were automatically segmented using Freesurfer 5.3,^1^ following the pipeline described at https://surfer.nmr.mgh.harvard.edu/fswiki/recon-all. Second, ^18^F-florbetaben PET images were co-registered to the 3D T1WI-labeled data using the Statistical Parametric Mapping Version 12 program (Wellcome Department of Imaging Neuroscience, University College, London, United Kingdom). Amyloid cortical SUVR was determined as the average of the standardized uptake value normalized by the uptake in the cerebellar gray matter, with this reference region being selected from the cerebellum external and cortex segments on MRI. Third, pattern analysis was conducted between Aβ present in the plasma and SUVR in amyloid PET using the coefficient of linear correlation. The results showed the average correlation coefficient in each brain region of interest as color-coded overlays on the representative MR image.

### Statistical Analyses

Continuous variables were compared using Student’s *t*-test and are presented as the mean ± SD. Categorical variables were compared using the chi-squared test or Fisher’s exact test. For multiple comparisons, we performed non-parametric statistical testing as the data were not normally distributed (Kruskal–Wallis one-way analysis of variance with the Dunn test for *post hoc* comparison between individual pairs). Multiple linear regression was performed using vascular risk factors (hypertension, diabetes mellitus, hyperlipidemia, and history of ischemic heart disease and stroke), *APOE* ε4 status, and Aβ_40_, Aβ_42_, and Aβ_42/40_ as independent variables. Statistical significance was set at *p* < 0.05, in two-tailed tests. Statistical analyses were performed using SPSS (version 20.0; IBM, Armonk, NY) and Prism 8 (GraphPad Software, San Diego, CA). Also, to compute the area under the curve (AUC), a receiver operator characteristic (ROC) curve was created using Prism 8. We used a hybrid Wilson/Brown method to compare ROC curves and determine the 95% confidence intervals associated with the sensitivities and specificities (at Youden index thresholds).

## Results

### Demonstrating the Platform’s Performance in Plasma Aβ Analysis

**FIGURE 1:**
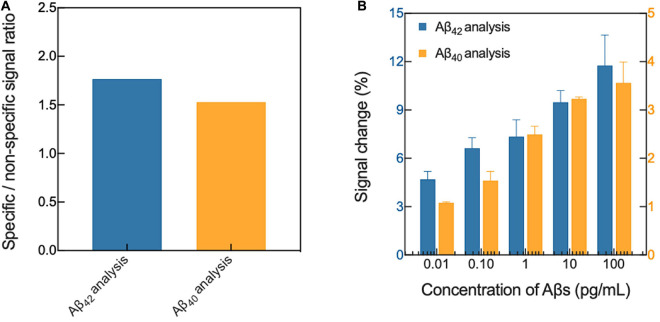
Verification of **(A)** selectivity and **(B)** sensitivity of the biosensor in 1 mM PBS. Aβ, amyloid-β; PBS, phosphate-buffered saline.

Our biosensing platform showed high specificity in the analysis of Aβs in 1 mM PBS, depending on the type of immobilized antibody on the surface of the platform (Figure 1A). Because the selectivity of the sensor indicates how well the sensor will respond specifically to the target analyte (Bhalla et al., 2016), we compared the output signal of the sensor in Aβ-specific and non-specific binding conditions. In the biosensor in which Aβ_42_-specific antibodies were immobilized, an approximately 1.862-fold higher signal change occurred following the specific binding of Aβ_42_ compared with the change following the non-specific binding of Aβ_40_ (approximately 5.928 ± 0.357% and 3.183 ± 0.318%, respectively; *p* < 0.0001). However, in the biosensor where Aβ_42_-specific antibodies were immobilized, the signal change due to the specific binding of Aβ_42_ was approximately 1.536-fold higher than that due to non-specific binding of Aβ_40_ (approximately 3.325 ± 0.430% and 2.165 ± 0.394%, respectively; *p* < 0.01). In addition, the average impedance variation due to buffer exchange and non-specific adsorption of plasma proteins on the surface of the platform was approximately 2.086 ± 0.585%.

The platform also had excellent sensitivity in the Aβ analysis, and the changes upon specific binding of Aβ_42_ and Aβ_4__0_ ranged from approximately 3.241 ± 0.053%−10.684 ± 1.296% and from 1.563 ± 0.169% to 3.919 ± 0.632%, respectively, depending on the concentrations of Aβ in 1 mM PBS (Figure 1B). In general, the sensitivity of a sensor is defined as the change in the output signal in the sensor as a function of the concentration of the analyte (S = Δsignal/Δanalyte), and is normally calculated from the slope of the linear calibration curve (Morales and Halpern, 2018). Therefore, through a linear regression analysis (linear equation: y = intercept + slope x, the value of the slope signifies the sensitivity of the sensor for Aβ binding), the sensitivities were calculated to be approximately 0.777 ± 0.106 and 1.036 ± 0.091 for Aβ_42_ and Aβ_40_ analysis, respectively. Furthermore, we demonstrated the possibility of highly sensitive analysis of Aβs spiked in standard plasma, and as a result, our sensing platform for the analysis of Aβs spiked in plasma was verified.

Furthermore, we verified that the impedance changes of our sensing platform ranged from approximately 3.627 ± 0.201% to 6.836 ± 0.657% and from 4.126 ± 0.369% to 8.476 ± 0.935%, respectively, depending on the concentrations of Aβ spiked in the plasma (Table 1). The results suggested that our sensing platform is useful for plasma Aβ analysis.

**TABLE 1:** Impedance changes according to the concentrations of Aβ spiked in the plasma.

Concentrations, pg/mL	Average impedance change, %	
	Aβ_40_	Aβ_42_
0.01	4.126 (0.369)	3.627 (0.201)
0.1	5.119 (0.102)	4.293 (0.513)
1	5.969 (0.472)	4.548 (0.588)
10	6.784 (0.574)	5.649 (0.655)
100	8.476 (0.935)	6.836 (0.657)

*Values are means (SD).*

*Aβ, amyloid-β.*

### Demographics and Plasma Aβ Analysis in Patients With Cognitive Decline

**FIGURE 2:**
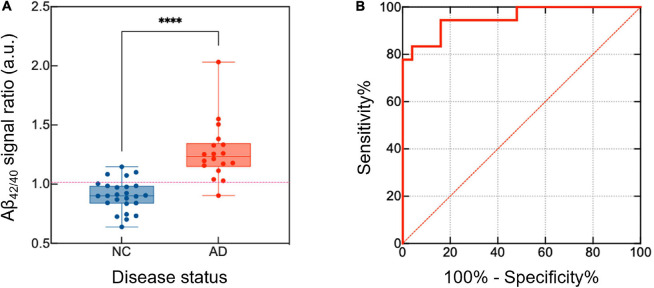
Differentiation of the NC and AD groups based on the analysis of plasma Aβ_42/40_ levels. To distinguish between the NC and AD groups, the relative signal level of Aβ_42_ and Aβ_40_ was calculated. **(A)** The ratio of the signal between the Aβ_42_ and Aβ_40_ was higher in the AD group than in the NC group. **(B)** A high AUC value was calculated using the ROC (AUC = 0.952, 95% CI, 0.892–1.000). *p*-values are indicated with asterisks: ****p < 0.0001. NC, normal control; AD, Alzheimer’s disease; Aβ, amyloid-β; AUC, area under the curve; ROC, receiver operating curve; CI, confidence interval.

Table 2 shows the demographics and plasma Aβ levels of the study participants. Based on amyloid PET positivity, participants were classified into a normal control (NC, *n* = 25) or AD (*n* = 19) group. The average signal levels of Aβ_40_ (*p* = 0.012) and Aβ_42_ (*p* = 0.004) were significantly different between the NC and AD groups. This difference was clearly reduced when the signal level of Aβ_42_ was divided by that of Aβ_40_ (NC vs. AD: *p* < 0.001 for Aβ_42/40_) (Figure 2A). In addition, the concentration of Aβs, estimated from the impedance changes based on the sensitivity curves of our sensing platform, were calculated (Supplementary Table 1). ROC curve analysis demonstrated that the ratio of Aβ_42/40_ was the best predictor of amyloid PET positivity, with the AUC of 0.952 [95% confidence interval (CI), 0.892–1.000] (Figure 2B). Participants with Aβ_42/40_ values exceeding 1.016 were considered as AD patients, as this value gave the maximum Youden index with a positive percentage agreement of 0.81 (95% CI, 0.75–0.96) and a negative percentage agreement of 0.95 (95% CI, 0.67–0.83) with amyloid PET positivity.

**TABLE 2:** Demographics and clinical characteristics of study participants.

	Total	NC	AD
N	44 (100.0)	25 (56.8)	19 (43.2)
Age, years	69.7 (8.0)	68.4 (6.5)	71.5 (9.5)
Female	31 (70.5)	17 (68.0)	14 (73.7)
Education, years	9.4 (5.6)	10.2 (6.2)	8.5 (4.7)
*APOE* ε4 status	18 (40.9)	8 (32.0)	10 (52.6)
Hypertension	27 (61.4)	18 (72.0)	9 (47.4)
Diabetes mellitus	15 (34.1)	8 (32.0)	7 (36.8)
Dyslipidemia	25 (56.8)	14 (56.0)	11 (57.9)
History of IHD	5 (11.4)	4 (16.0)	1 (5.3)
History of stroke	8 (18.2)	4 (16.0)	4 (21.1)
Cognitive status			
SCD	20 (45.5)	20 (80.0)	–
aMCI	7 (15.9)	5 (20.0)	2 (10.5)
Dementia	17 (38.6)	–	17 (89.5)
MMSE	21.8 (56.0)	25.1 (4.6)^a^	17.5 (4.8)
Amyloid PET SUVR	1.393 (0.259)	1.198 (0.059)^a^	1.649 (0.182)
Plasma Aβ, fg/mL			
Aβ_40_	219.9 (534.5)	343.8 (676.8)	56.9 (110.2)
Aβ_42_	10,134 (5,095.1)	1,117 (2,798.6)	21,998 (66,717.8)
Plasma Aβ, %			
Aβ_40_	4.520 (0.967)	4.846 (0.906)^a^	4.109 (0.881)
Aβ_42_	4.641 (0.871)	4.320 (0.733)^a^	5.063 (0.858)
Aβ_42/40_ (a.u.)	1.059 (0.256)	0.904 (0.127)^a^	1.263 (0.238)

*Values are means (SD) or N (%).*

*The value of plasma Aβ (%) is the average impedance change of the biosensing platform.*

*Statistical analyses were performed using the chi-squared test, Fisher’s exact test, or Student’s t-test.*

*^a^Difference between NC and AD, p < 0.05.*

*NC, normal controls; AD, Alzheimer’s disease; N, number; APOE, apolipoprotein E; IHD, ischemic heart disease; SCD, subjective cognitive decline; aMCI, amnestic mild cognitive impairment; MMSE, Mini-Mental State Examination; PET, positron emission tomography; SUVR, standardized uptake value ratio; Aβ, amyloid-β.*

### Neuroimaging Pattern Analysis Between Plasma Aβ_42/40_ Levels and Amyloid Positron Emission Tomography Standardized Uptake Value Ratio

**FIGURE 3:**
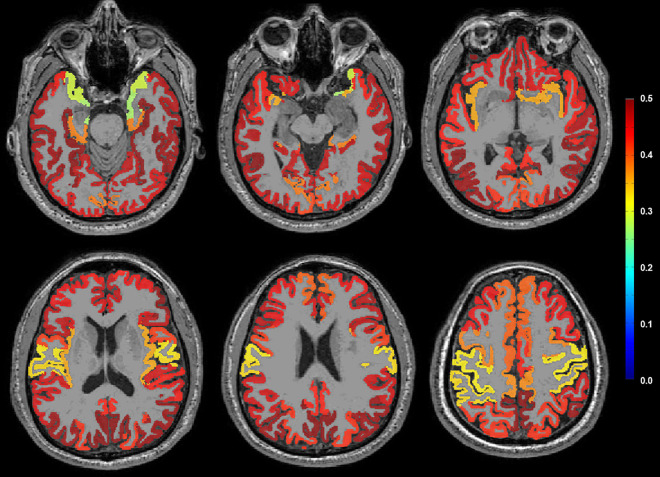
Correlation coefficient (*r*) between plasma Aβ_42/40_ and amyloid PET SUVR on representative MRI using pattern analysis. A moderate positive correlation (red color) was observed in the bilateral frontal, parietal, temporal, and occipital cortices, except the primary motor and sensory cortices. Aβ, amyloid-β; SUVR, standardized uptake value ratio.

A topographical pattern of the correlation coefficient (*r*) between plasma Aβ_42/40_ and amyloid PET SUVR is presented in Figure 3. Plasma Aβ_42/40_ and amyloid PET SUVR showed moderate positive correlations in the bilateral frontal, parietal, temporal, and occipital cortices, except for the primary motor and sensory cortices, suggesting a typical pattern of regional deposition for neocortical Aβ in AD.

### Neuropsychological Performance in the Normal Control and Alzheimer’s Disease Groups

As shown in Table 3, we compared neuropsychological performance results between the NC and AD groups. Significant differences were observed in the mean Z-scores of the K-BNT (*p* = 0.002), SVLT [immediate recall (*p* < 0.001), delayed recall (*p* < 0.001), recognition (*p* < 0.001)], RCFT [copy (*p* = 0.028), immediate recall (*p* < 0.001), delayed recall (*p* < 0.001), recognition (*p* = 0.001)], COWAT phonemic total (*p* = 0.030), and Stroop color reading (*p* = 0.012) tasks between the NC and AD groups.

**TABLE 3:** Neuropsychological performance in the NC and AD groups.

	NC	AD	*p*-value
**Attention**			
Digit span forward	–0.41 (0.82)	–0.41 (1.06)	0.995
Digit span backward	–0.35 (0.96)	–0.91 (1.01)	0.067
**Language**			
K-BNT	–0.66 (1.36)	–2.66 (2.55)	0.002
**Visuospatial function**			
RCFT copy	0.03 (1.08)	–3.36 (6.03)	0.028
**Verbal memory**			
SVLT immediate recall	–0.86 (0.82)	–2.05 (1.16)	<0.001
SVLT delayed recall	–0.86 (0.72)	–2.34 (0.96)	<0.001
SVLT recognition	–0.16 (1.10)	–2.61 (2.23)	<0.001
**Visual memory**			
RCFT immediate recall	–0.40 (0.92)	–1.71 (0.95)	<0.001
RCFT delayed recall	–0.48 (0.96)	–1.92 (0.89)	<0.001
RCFT recognition	–0.37 (0.86)	–2.14 (1.96)	0.001
**Frontal/executive function**			
COWAT phonemic total	–0.58 (1.00)	–1.43 (1.26)	0.030
Stroop color reading	–0.22 (1.12)	–2.68 (2.97)	0.012

*Age- and education-specific Z-scores were used to compare neuropsychological performance between the NC and AD groups.*

*NC, normal controls; AD, Alzheimer’s disease; K-BNT, Korean version of the Boston Naming Test; RCFT, Rey-Osterrieth Complex Figure Test; SVLT, Seoul Verbal Learning Test; COWAT, Controlled Oral Word Association Test.*

### Association Between the Level of Plasma Aβs and Neuropsychological Test Results

The results of multivariate regression analysis between the levels of plasma Aβ (Aβ_40_, Aβ_42_, and Aβ_42/40_) and neuropsychological performance are presented in Table 4. There were no significant correlations between plasma Aβ_40_ and Aβ_42_ levels and neuropsychological performance in the study participants. However, higher levels of plasma Aβ_42/40_ showed a significant negative correlation with the Z-score of the SVLT [immediate recall (*p* = 0.014), delayed recall (*p* = 0.006), recognition (*p* = 0.018)], and RCFT [immediate recall (*p* = 0.042), delayed recall (*p* = 0.048), recognition (*p* = 0.005)] tasks in the study participants.

**TABLE 4:** Correlations of plasma Aβ_40_, Aβ_42_, and Aβ_42/40_ levels with the results of neuropsychological tests.

	Plasma Aβ_40_			Plasma Aβ_42_			Plasma Aβ_42/40_		
	*B*	SE	*p*-value	*B*	SE	*p*-value	*B*	SE	*p*-value
**Attention**									
Digit span forward	–0.17	0.15	0.237	0.00	0.16	0.998	0.63	0.56	0.267
Digit span backward	0.05	0.17	0.754	–0.18	0.17	0.310	–0.84	0.63	0.191
**Language**									
K-BNT	0.25	0.36	0.494	–0.24	0.38	0.529	–2.17	1.36	0.121
**Visuospatial function**									
RCFT copy	0.93	0.75	0.223	0.53	0.81	0.517	–1.43	2.96	0.632
**Verbal memory**									
SVLT immediate recall	0.32	0.18	0.078	–0.09	0.19	0.648	–1.69	0.66	0.014
SVLT delayed recall	0.35	0.19	0.078	–0.19	0.21	0.371	–2.02	0.69	0.006
SVLT recognition	0.59	0.35	0.098	–0.13	0.38	0.727	–3.19	1.28	0.018
**Visual memory**									
RCFT immediate recall	0.19	0.20	0.353	–0.22	0.21	0.301	–1.55	0.73	0.042
RCFT delayed recall	0.11	0.21	0.611	–0.33	0.22	0.135	–1.58	0.77	0.048
RCFT recognition	0.54	0.28	0.057	–0.25	0.30	0.413	–3.00	1.01	0.005
**Frontal/executive function**									
COWAT phonemic total	0.41	0.24	0.105	–0.17	0.27	0.525	–1.78	0.98	0.078
Stroop color reading	0.69	0.43	0.120	–0.01	0.49	0.980	–2.47	1.61	0.139

*Multiple linear regression was performed using vascular risk factors (hypertension, diabetes mellitus, hyperlipidemia, and history of ischemic heart disease and stroke), APOE ε4 status, and Aβ_40_, Aβ_42_, and Aβ_42/40_ as independent variables.*

*Aβ, amyloid-β; K-BNT, Korean version of the Boston Naming Test; RCFT, Rey-Osterrieth Complex Figure Test; SVLT, Seoul Verbal Learning Test; COWAT, Controlled Oral Word Association Test; APOE, apolipoprotein E.*

## Discussion

In this study, we investigated the association between plasma Aβ levels and neuropsychological performance in patients with cognitive decline using a highly sensitive nano-biosensing platform fabricated by combining micro/nanotechnology with DEP. We confirmed that our methods for precise AD diagnosis were reliable for the selective analysis of extremely low levels of plasma Aβ, and the neuroimaging pattern analysis supported this result. Unexpectedly, higher levels of plasma Aβ_42/40_ were negatively associated with memory performance in patients with cognitive decline, but not in all cognitive domains. Our results demonstrated that plasma Aβ analysis using a nano-biosensing platform could be a useful tool for diagnosing AD and assessing memory performance in patients with cognitive decline.

We found that the ratio of plasma Aβ_42_ to Aβ_40_ (Aβ_42/40_) was the best predictor of amyloid PET positivity in the study participants, and our findings were consistent with previous results using plasma Aβ analysis (Lue et al., 2017; Park et al., 2017; de Rojas et al., 2018). Our results are also in line with the fact that the combination of Aβ_40_ and Aβ_42_ is more reliable in predicting the risk of developing AD than either biomarker individually (van Oijen et al., 2006). With the remarkable advances in technology to date, there have been efforts to detect Aβ in the CSF and blood using various technical methods (Wang et al., 2020). However, since Aβ levels are approximately 10 times lower in the plasma than in CSF despite the overall protein content being 100-fold higher, the occurrence of strong interference has been raised as a key problem to overcome in plasma Aβ analysis with conventional bioassay systems (Kleinschmidt et al., 2016; Wang et al., 2020). In fact, the concentrations of Aβ in the CSF are present at the level of sub-ng/mL or several ng/mL, while those in the blood are at much lower levels of pg/mL (Yang et al., 2017). This means that the required limit of detection (LoD) for assaying Aβ protein in human blood should be several pg/mL. Previous attempts to demonstrate blood-based AD biomarkers using an enzyme-linked immunosorbent assay (ELISA) have been unsuccessful because the concentration of Aβ is ultralow in AD and the performance of horseradish peroxidase used in ELISA is highly dependent on pH and temperature (Jiao et al., 2018; Lyu et al., 2020). Recently, mass spectrometry-based AD biomarker testing has also been developed to diagnose AD through high-performance measurement of plasma Aβ (Nakamura et al., 2018; Oeckl and Otto, 2019). However, as far as we know, blood-based Aβ assay technologies using ELISA and mass spectrometry demonstrate LoD values in the hundreds of pg/mL (Sehlin et al., 2010; Oeckl and Otto, 2019). In addition, recent studies related to blood-based AD diagnosis have reported that digital ELISA technology, or the single molecule assay (SIMOA), can detect specific N-terminal Aβ peptides in plasma with high accuracy (De Meyer et al., 2020; Thijssen et al., 2021). Immunomagnetic reduction (IMR) technology has also been introduced as a promising blood-based biomarker platform to diagnose AD at early stages (Yang et al., 2017; Lue et al., 2019). IMR has technical advantages over SIMOA: SIMOA use magnetic beads to purify target molecules and this process usually causes loss of target molecules (Yang et al., 2017). However, IMR technology and our nano-biosensing platform both directly measure target molecules using a primary antibody. Furthermore, our platform has the advantage of detecting small biomolecules by precisely detecting changes in electrical signals and maximizing them using DEP (Kim et al., 2019). When Aβ reacts with a specific antibody immobilized on the sensor surface, the electric field formed between the electrodes is affected by Aβ, thus changing the electrical signal of the sensor. Additionally, DEP causes a filtration effect, which enriches Aβ present at extremely low levels in the plasma, enabling a sensitive LoD that can detect the concentrations of plasma Aβ in the range of fg/mL with high accuracy. Using this technology, we could precisely quantify the level of plasma Aβ, including its monomeric form. We suggest that our method is a promising screening tool for identifying AD as a first-line clinical evaluation.

Our major finding in the present study was that plasma Aβ_42/40_ was negatively correlated with memory performance in patients with cognitive decline. The AD group showed poorer neuropsychological performance than the NC group in all cognitive domains except attention. However, in multivariate regression analysis, upon inclusion of age, education, vascular risk factors, and *APOE* ε4 status, higher levels of plasma Aβ_42/40_ showed significant negative correlations with verbal and visual memory performance, including immediate recall, delayed recall, and recognition tests. Therefore, we suggest that the biosensing platform used in this study sensitively reflects the degree of memory impairment in the study participants. Since the introduction of new research frameworks using Aβ, tau, and neurodegeneration, the definition of AD has shifted from a syndromal to a biological construct based on biomarkers that are proxies of pathology (Jack et al., 2018). Accordingly, most research has mainly focused on the application of molecular (PET) and structural (MRI) imaging in the diagnosis of AD. Although remarkable progress has been made in blood-based diagnostic methods for AD, it is unclear whether these methods reflect neuropsychological performance in patients with cognitive decline in clinical settings. Furthermore, most studies using blood-based AD biomarkers have only reported the diagnostic power for the distinction between the NC and AD groups. However, to the best of our knowledge, there have been no reports on which cognitive domains significantly correlate with biomarkers.

Since amnestic syndrome is a typical presentation of AD onset (Cerami et al., 2017), we suggest that our findings in this study have clinical significance in terms of the precise diagnosis of AD and the clinical assessment of memory impairment in patients with cognitive decline. We speculate that there are several explanations for the reason that only the memory domain was negatively correlated with the level of plasma Aβ_42/40_ in this study. Since our biosensing platform was able to detect the monomers and oligomers among the various forms of Aβ in the blood, we suggest that our precise method could more accurately reflect differences in memory impairment, a core feature of AD, between the NC and AD groups. Alternatively, as various factors that could affect cognitive function even in normal aging were statistically mitigated (Salthouse, 2009; Lee et al., 2018), only the level of plasma Aβ_42/40_ and memory domain may have shown a significant association.

This study has several limitations. First, since our study was conducted using blood samples collected from a cross-sectional study, further validation studies are needed. Second, the statistical power of our analyses was relatively low due to the small sample size. Third, since our method measured the total amount of Aβ present in the plasma (mainly monomers but also oligomers consisting of 10 or fewer monomers with a molecular weight of 3–10 kDa), it was not possible to evaluate the effect of each form on the study results. Finally, our study participants were enrolled from a single memory clinic, which might limit the generalizability of the results. Despite these limitations, we suggest that our study might have clinical significance because plasma Aβ analysis using a highly sensitive nano-biosensing platform could not only be a useful non-invasive method for diagnosing AD, but also showed a significant association with memory performance in patients with cognitive decline. Further investigations with a larger sample size are necessary to clarify the possible relevance of plasma Aβ as a blood-based biomarker for precise AD diagnosis.

## Data Availability Statement

The datasets generated for this study are available on request to the corresponding authors.

## Ethics Statement

The studies involving human participants were reviewed and approved by the Institutional Review Board of Kyung Hee University Hospital. The patients/participants provided their written informed consent to participate in this study.

## Author Contributions

IH and JL: conception, design of the study, and final approval of the manuscript. GY, HK, KL, IH, HR, SY, K-CP, and JL: acquisition of data. GY, HK, H-GK, KL, IH, HR, G-HJ, SY, K-CP, IH, and JL: analysis and interpretation of the data. GY, HK, HR, K-CP, IH, and JL: drafting and revising the manuscript for content. All authors contributed to the article and approved the submitted version.

## Conflict of Interest

The authors declare that the research was conducted in the absence of any commercial or financial relationships that could be construed as a potential conflict of interest.

## Publisher’s Note

All claims expressed in this article are solely those of the authors and do not necessarily represent those of their affiliated organizations, or those of the publisher, the editors and the reviewers. Any product that may be evaluated in this article, or claim that may be made by its manufacturer, is not guaranteed or endorsed by the publisher.

## Footnotes

1
http://surfer.nmr.mgh.harvard.edu/
