# Supplementary material for: Association Between Plasma Amyloid-β and Neuropsychological Performance in Patients With Cognitive Decline
Source: Front Aging Neurosci. 2021 Oct 25;13:736937. doi: 10.3389/fnagi.2021.736937 (PMC8573146; doi:10.3389/fnagi.2021.736937)
Supplement: Supplementary file 1 [file Data_Sheet_1.docx]

Association between plasma amyloid-β and neuropsychological performance in patients with cognitive decline

**Gyihyaon Yun^1†^, Hye Jin Kim^2†^, Hyug-Gi Kim^3^, Kyung Mi Lee^3^, Il Ki Hong^4^,** **Sang Hoon Kim^5^, Hak Young Rhee^6^, Geon-Ho Jahng^7^, Sung Sang Yoon^1^, Key-Chung Park^1^, Kyo Seon Hwang^2^*, and Jin San Lee^1^***

Departments of ^1^Neurology, ^3^Radiology, and ^4^Nuclear Medicine, and ^5^Otorhinolaryngology, Head and Neck Surgery, Kyung Hee University Hospital, Kyung Hee University College of Medicine, Seoul, Republic of Korea

^2^Department of Clinical Pharmacology and Therapeutics, Kyung Hee University College of Medicine, Seoul, Republic of Korea

Departments of ^6^Neurology and ^7^Radiology, Kyung Hee University Hospital at Gangdong, Kyung Hee University College of Medicine, Seoul, Republic of Korea

^†^These authors contributed equally to this work.

*** Correspondence:**Jin San Lee, MD, PhD
xpist@naver.com

Kyo Seon Hwang, PhD

k.hwang@khu.ac.kr

**Running Title:** Plasma Amyloid-β and Neuropsychological Performance

**Supplementary data:** Supplementary Methods, Supplementary Results (Supplementary Table 1)

# Supplementary Methods

## Reagents and Materials

Sulfuric acid (H_2_SO_4_, 98%), hydrogen peroxide (H_2_O_2_, 35%), and isopropyl alcohol (IPA, 99.7%) were purchased from Daejung Chemical & Metals Co. Ltd. (Gyeonggi-do, Korea). 3-(ethoxydimethylsilyl) propylamine (APMES, 97%), *N*-(3-dimethyl aminopropyl)-*N*-ethylcarbodiimide hydrochloride (EDC, molecular weight [MW]: 155.249 g/mol), *N*-hydroxysuccinimide (NHs, MW: 115.09 g/mol), and glutaraldehyde solution (50 wt%) were purchased from Sigma-Aldrich (St. Louis, MO). We purchased 100 mM phosphate-buffered saline (PBS, pH 7.4) from Corning Korea Co. Ltd., (Seoul, Korea), and 0.2 M 2-morpholinoethanesulfonic acid monohydrate (MES, pH 5.5) buffer from Thermo Fisher Scientific (Waltham, MA). Standard human plasma was purchased from Sigma-Aldrich (cat. no. P9525). Polydimethylsiloxane (SYLGARD 184 A/B) was purchased from Dowhitech Silicone Co. Ltd. (Gyeonggi-do, Korea). All reagents and solvents were used as received without further purification. The distilled water used in all experiments had a resistivity higher than 18 MΩ·cm^-1^ from the AquaMAX Ultra 370 series (Young In Chromass Co., Gyeonggi-do, Korea). Aβ protein fragments 1–40 (Aβ_40_) and 1-42 (Aβ_42_), with a MW of approximately 4.5 kDa and human hemoglobin were purchased from Sigma-Aldrich. Purified anti-Aβ 1–40 (11A50–B10) antibody and purified anti-Aβ 1–42 (12F4) antibody, which were specific for the isoform ending at the 40^th^ and 42^nd^ amino acids of Aβ_42_, respectively, were purchased from BioLegend (San Diego, CA).

# Supplementary Results

## Estimations of plasma Aβ levels in the NC and AD groups

**Supplementary Table 1** Estimations of plasma Aβ levels in the NC and AD groups

| **Levels of Aβ**  **in the NC group’s plasma [fg/mL]** | | | | **Levels of Aβ**  **in the AD group’s plasma [fg/mL]** | | | |
| --- | --- | --- | --- | --- | --- | --- | --- |
| **Sample #** | **Aβ_40_** | **Aβ_42_** | **Aβ_42/40_** | **Sample #** | **Aβ_40_** | **Aβ_42_** | **Aβ_42/40_** |
| 1 | 64.40 | 715.5 | 11.11 | 1 | 11.31 | 534.1 | 47.22 |
| 2 | 1.970 | 17.45 | 8.86 | 2 | 34.60 | 5,434 | 157.05 |
| 3 | 73.37 | 266.0 | 3.63 | 3 | 66.32 | 81,204 | 1224.43 |
| 4 | 3,024 | 807.9 | 0.27 | 4 | 155.7 | 3.485 | 0.02 |
| 5 | 56.73 | 474.6 | 8.37 | 5 | 0.456 | 2,911 | 6383.77 |
| 6 | 8.361 | 6.914 | 0.83 | 6 | 482.3 | 294,819 | 611.28 |
| 7 | 16.71 | 34.71 | 2.08 | 7 | 0.338 | 36.52 | 108.05 |
| 8 | 82.08 | 91.73 | 1.12 | 8 | 5.034 | 366.7 | 72.84 |
| 9 | 59.16 | 13.36 | 0.23 | 9 | 1.803 | 757.9 | 420.35 |
| 10 | 4.741 | 6.824 | 1.44 | 10 | 62.51 | 6,301 | 100.80 |
| 11 | 630.7 | 11,239 | 17.82 | 11 | 1.552 | 52.83 | 34.04 |
| 12 | 817.9 | 1,078 | 1.32 | 12 | 18.22 | 8,811 | 483.59 |
| 13 | 3.877 | 12.06 | 3.11 | 13 | 39.09 | 1,672 | 42.77 |
| 14 | 228.6 | 11.19 | 0.05 | 14 | 29.46 | 415.1 | 14.09 |
| 15 | 216.6 | 694.9 | 3.21 | 15 | 4.258 | 126.0 | 29.59 |
| 16 | 11.51 | 188.8 | 16.40 | 16 | 151.5 | 9,059 | 59.80 |
| 17 | 6.328 | 71.40 | 11.28 | 17 | 4.296 | 6.520 | 1.52 |
| 18 | 58.85 | 433.7 | 7.37 | 18 | 11.72 | 1,663 | 141.89 |
| 19 | 1,639 | 9,792 | 5.97 | 19 | 2.137 | 314.9 | 147.36 |
| 20 | 3.390 | 3.847 | 1.13 |  | | | |
| 21 | 21.04 | 1,026 | 48.76 |  |  |  |  |
| 22 | 104.9 | 185.7 | 1.77 |  |  |  |  |
| 23 | 131.5 | 26.47 | 0.20 |  |  |  |  |
| 24 | 182.1 | 588.4 | 3.23 |  |  |  |  |
| 25 | 1,147 | 138.2 | 0.12 |  |  |  |  |

Aβ, amyloid-β; NC, normal control; AD, Alzheimer’s disease.
